# Thermodynamics of a Compressible Maier-Saupe Model Based on the Self-Consistent Field Theory of Wormlike Polymer

**DOI:** 10.3390/polym9020048

**Published:** 2017-02-04

**Authors:** Ying Jiang, Cristina Greco, Kostas Ch. Daoulas, Jeff Z. Y. Chen

**Affiliations:** 1 School of Chemistry and Environment, Center of Soft Matter Physics and its Applications, Beihang University, Beijing 100191, China; yjiang@buaa.edu.cn; 2 Max Planck Institute for Polymer Research, Ackermannweg 10, 55128 Mainz, Germany; greco@mpip-mainz.mpg.de; 3 Department of Physics and Astronomy, University of Waterloo, Waterloo, ON N2L 3G1, Canada

**Keywords:** self-consistent field theory, polymers, liquid crystals, Maier–Saupe, nematics

## Abstract

This paper presents a theoretical formalism for describing systems of semiflexible polymers, which can have density variations due to finite compressibility and exhibit an isotropic-nematic transition. The molecular architecture of the semiflexible polymers is described by a continuum wormlike-chain model. The non-bonded interactions are described through a functional of two collective variables, the local density and local segmental orientation tensor. In particular, the functional depends quadratically on local density-variations and includes a Maier–Saupe-type term to deal with the orientational ordering. The specified density-dependence stems from a free energy expansion, where the free energy of an isotropic and homogeneous homopolymer melt at some fixed density serves as a reference state. Using this framework, a self-consistent field theory is developed, which produces a Helmholtz free energy that can be used for the calculation of the thermodynamics of the system. The thermodynamic properties are analysed as functions of the compressibility of the model, for values of the compressibility realizable in mesoscopic simulations with soft interactions and in actual polymeric materials.

## 1. Introduction

Strategies for modelling polymeric liquid crystals (PLC) present significant interest, in view of several important applications, including high-strength fibres [1], stimuli-responsive elastomers [2,3], meta-materials [4,5], biomedical applications [6] and organic-electronics [7,8,9]. The topic itself links to a number of fundamental questions in statistical physics, materials science and polymer physics. The spectrum of theoretical approaches that can be taken to model PLC is broad: from all-atom and coarse-grained computer simulations to analytical approaches based on partition function calculation.

Currently, all-atom computer simulations of liquid-crystalline (LC) mesophases consisting of long polymer chains are impractical due to the large system sizes that must be addressed and the protracted equilibration times required; for a detailed discussion, see topical review in [10]. So far, atomistic simulations have focused on oligomer systems only, frequently by pre-assembling or directing the molecules into the LC mesophase, as illustrated by studies reported in [11,12,13,14,15]. Beyond the atomistic scale, coarse-grained (CG) models offer an alternative, reducing the degrees of freedom by representing groups of atoms in PLC chains [16,17,18,19,20] with single effective interaction centres. Progress has been made by employing CG models that are systematically developed [21,22,23] utilising information available from moderate, all-atom simulations. At the same time, these models are still quite close to the original microscopic structure so that their performance is restricted by features, such as hard excluded volume constraints. Therefore, a powerful strategy to study the long wavelength properties of PLC (i.e., on scales comparable or larger than the size of the polymer chains) is to employ “soft” or “mesoscopic” models with the interactions of the order of magnitudes comparable to the thermal energy, $k_{B}T$. The reduced strength of interactions effectively captures the large number of microstates, which are projected on a single CG configuration [23,24,25,26,27].

A systematic development of a mesoscopic model inevitably involves approximations made to average over atomistic-scale configurations. Furthermore a “bottom-up” approach is seriously affected by imperfections in atomistic force-fields or equilibration procedures used to obtain the reference data. Thus, mesoscale theories and simulations of PLC benefit from “top-down” strategies, in which some ingredients of the models are introduced phenomenologically, to reproduce selected material properties by construction.

A minimal representation of the basic molecular architecture, suitable for LC polymers, is the wormlike-chain (WLC) model. This model incorporates two basic scales characterising the chain, one of which is microscopic (persistence length), while the other is mesoscopic (contour length). The WLC model allows one to specify explicitly segmental orientations and therefore can be easily incorporated into anisotropic “top-down” non-bonded potentials. In addition to particle-based computer simulations, the statistical mechanics of WLC-based models can be investigated within the self-consistent field theory (SCFT) approximation. In SCFT, the segment-segment interactions are replaced by background mean fields, acting on a single wormlike polymer. These mean fields are in turn linked to averaged conformations and distribution of chains in space, self-consistently. The theoretical framework can straightforwardly produce the thermodynamic properties, such as the free energies, which are difficult to obtain in particle-based computer simulations. Theoretical and technical aspects of SCFT based on the Gaussian-chain formalism have been extensively discussed in the literature where textbooks and original derivations can be found [28,29,30,31,32,33,34,35,36]. Comparing to the Gaussian chain, the SCFT formalism for wormlike chains must include the orientational dependence and hence has a more complicated structure. The early development of the formalism can be traced back to [37]. Subsequently, a generalised version suitable for studying liquid-crystal systems consisting of homopolymers was developed for Onsager-type interactions [38,39,40,41], where the density of the system alone is an important factor driving the formation of the nematic state.

SCFT schemes based on MS-type (Maier–Saupe) models have been applied to a broad range of systems: from solutions and melts of homopolymers [42,43,44,45], to nanostructured block-copolymer melts [46,47,48,49,50,51,52,53,54]. Currently there is an increased interest in employing mean-field approaches based on MS-type models for the description of nematic mesophases of conjugated polymers (e.g., see [9,15,55,56]). Despite these efforts, there are important aspects of thermodynamics of MS-type models, which have not been sufficiently explored. For instance, most SCFT studies consider incompressible MS-type models. Therefore, the basic question of how compressibility interferes with other thermodynamic properties, such as the free energy landscape, isotropic/nematic transition and the strength of nematic ordering, has received almost no attention. Understanding the compressibility effects in MS-type models is particularly important, because particle-based simulations can operate only with compressible versions, in contrast with SCFT [56,57,58]. In these mesoscale simulations, the thermal compressibility $k_{T}$ of polymer nematics is large, exceeding by one or two orders of magnitude the compressibility of actual polymers.

Here, motivated by this question, we perform an SCFT study of the thermodynamics of polymer nematics described by a compressible MS-type model, in which the MS anisotropic non-bonded interaction is augmented by an isotropic, repulsive potential. This pairwise interaction is equivalent to a simple constraint on density variations introduced initially by Helfand and Tagami in SCFT studies of polymer blends [59]. By varying the strength of the repulsion, we can systematically reduce the compressibility of the model switching from situations typically encountered in mesoscale simulations to values representative of real materials. On the one hand, a basic limitation of SCFT is the negligence of density and orientational fluctuations, as well as density correlations in liquid packing. On the other hand, for a first systematic study of the compressible MS model, these approximations are advantageous since they allow for simplified insights into how different parameters affect the PLC thermodynamics. Importantly, comparing to particle-based simulations, SCFT provides a mechanism to reach the limit of a fully-incompressible system.

Our manuscript is organised as follows. In Section 2, we present an overview of the compressible MS-type model based on the WLC. The final expressions obtained via SCFT for the free energy landscape, equations of state and phase equilibria are presented in Section 3. In doing so, we hope to make our study accessible to a broader audience, which might be rather interested in the physics of the problem and not in the details of the formalism. These details are discussed in Appendix A and Appendix B. In Section 4, Section 5 and Section 6, we present a simple example focusing on the thermodynamics and transition properties of the model, paying special attention to the effects of compressibility. With realistic physical parameters that can be traced back to experimental systems, we calculate isobaric properties, which show a crossover from a compressible system to incompressible system. We conclude by remarks and a brief summary, linking our findings to related topics.

## 2. Compressible MS Model of Wormlike Chains

### 2.1. Backbone Wormlike Chain

**Figure 1:**
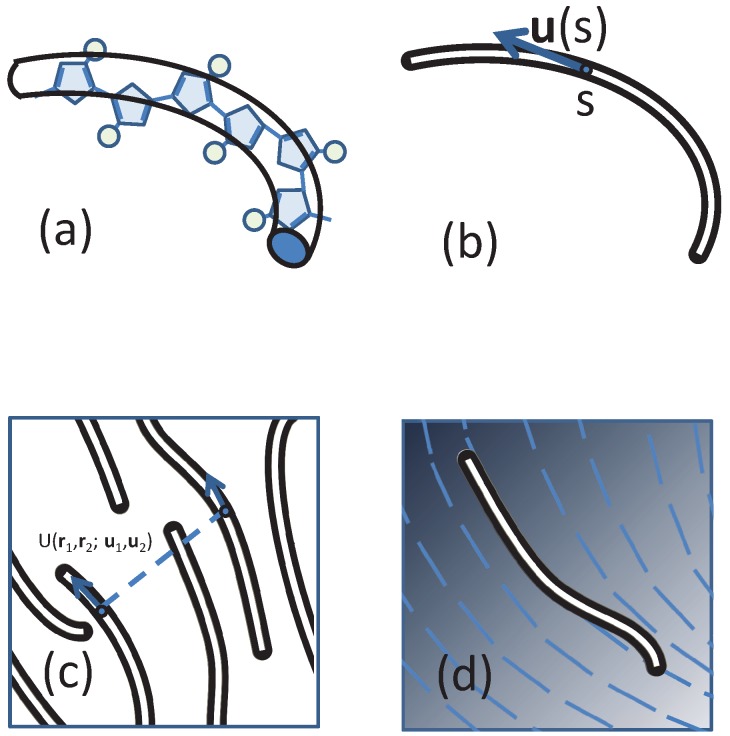
Sketches of (**a**) approximating a real polymer by a backbone wormlike chain; (**b**) the definition of the tangent direction of a polymer segment; (**c**) compressible polymers that have a direction ordering and (**d**) the concept of self-consistent field theory (SCFT), which treats the monomer-monomer interaction by a directionally-dependent background field, illustrated by blue lines.

The compressible MS model considered in our study is consistent with an approach recently introduced [56,57,58,60] for mesoscale particle-based modelling of nematic mesophases in polymers relevant to organic electronics. LC mesophases are formed [7,8,9] in several families of conjugated polymers (e.g., polyalkylthiophenes, polyfluorenes and PBTTs), which typically have a “hairy-rod” architecture, comprising a semiflexible backbone of bulky aromatic units with attached side-chains (represented in Figure 1a by circles).

The configuration of the backbone of a semiflexible polymer is dominated by a WLC Hamiltonian, $H_{b}$. For a system of volume *V* containing *n* homopolymers, the continuum version of the Hamiltonian is given by:

(1) $$\frac{H_{b}}{k_{B}T}=\frac{L_{p}}{2}\sum_{i=1}^{n}\int_{0}^{L}ds\;{\left|\frac{du_{i}\left(s\right)}{ds}\right|}^{2}.$$

In this expression, the configuration of the *i*-th WLC chain is represented by a continuous space curve with coordinate $R_{i}\left(s\right)$ [61], where the variable *s* corresponds to the arc length of the curve between the segment location and the starting terminal of a polymer. The local tangent vector is specified by the unit vector $u_{i}\left(s\right)\equiv dR_{i}\left(s\right)/ds$ (see Figure 1b). The bare persistence length $L_{p}$ describes the distance along the contour of a worm-like chain over which orientational correlations decay exponentially without an external potential in three dimensions [62]; the total contour length of a polymer chain is *L*. The Hamiltonian has been reduced by a factor where $k_{B}$ is the Boltzmann constant and *T* is the temperature.

### 2.2. Non-Bonded Interactions in a Compressible System

Within models used in particle-based simulations, non-bonded interactions between individual particles are introduced through potentials (typically pairwise). Here, following a common practice in field-theoretical schemes, we introduce the non-bonded interactions through a functional of collective degrees of freedom. We note that in certain examples (e.g., the Edwards model) the functionals in field-theoretical descriptions can be traced back to simple, pairwise potentials. This reduction is not possible in the most general case. Therefore, conceptually, defining interactions on the level of collective degrees of freedom should be seen as a special “top-down” coarse-graining strategy, where the functionals are introduced depending on the thermodynamic behaviour one aims to investigate. In our case, the functional must take into account that the underlying non-bonded interactions between segments depend on their orientation (Figure 1c).

We are mainly concerned about the density variations and the orientational ordering in the system. Due to the finite compressibility, the local density $\hat{\rho }\left(r\right)$ can vary. To quantify these variations, we introduce a volume fraction $\hat{\phi }\left(r\right)=\hat{\rho }\left(r\right)/\rho_{0}$ where $\rho_{0}$ is the segment number density of a uniform reference state corresponding to an isotropic melt. The configuration-dependent volume fraction is then expressed through the coordinates of individual segments as:

(2) $$\hat{\phi }\left(r\right)=\frac{N}{\rho_{0}}\sum_{i=1}^{n}\int_{0}^{1}dt\delta \left[r-R_{i}\left(t\right)\right].$$

Note that $\hat{\phi }\left(r\right)$ is normalized, such that:

(3) $$\int dr\hat{\phi }\left(r\right)=nN/\rho_{0}.$$

In the reference state, one has $\hat{\phi }\left(r\right)=1$. A typical wormlike-polymer chain is assumed to have *N* monomers, each representing a polymer segment of length *b* such that N=L/b. The effective parameter *b* does not enter into our theory explicitly, but the readers are reminded that some physical parameters defined below are related to the “per-monomer” concept.

The orientational properties are measured by a local second-rank tensor, defined through:

(4) $$\hat{S}\left(r\right)=N\rho_{0}^{-1}\sum_{i=1}^{n}\int_{0}^{1}dt\delta \left[r-R_{i}\left(t\right)\right]Q\left[u_{i}\left(t\right)\right].$$

The second-rank alignment tensor function Q(u) has 3×3 elements,

(5) $$Q\left(u\right)=\frac{3}{2}uu-\frac{I}{2}$$

in vector notation, where I is the unit tensor. Note that because we are dealing with a compressible system where the average of $\hat{\phi }$ can deviate away from unity, in a homogeneous system, the average of $\hat{S}$ is the product between the averaged Q and the average volume fraction.

In the current model, the non-bonded interactions are decomposed into isotropic and anisotropic contributions. The isotropic part can be viewed as the excess free energy of an isotropic, but positionally-inhomogeneous homopolymer melt. Expanded up to second order about the homogeneous reference state with density $\rho_{0}$, the Hamiltonian has the form:

(6) $$\begin{array}{cc} \frac{H_{\mathrm{iso}}}{k_{B}T}= & \int drf_{0}+\frac{\rho_{0}\mu_{0}\left(\rho_{0}\right)}{k_{B}TN}\int dr\left(\hat{\phi }\left(r\right)-1\right)+\\ & \frac{1}{2}\underset{\kappa \rho_{0}}{\underset{︸}{\left[\frac{1}{k_{B}Tk_{T}\left(\rho_{0}\right)}-\frac{\rho_{0}}{N}\right]}}\int dr{\left(\hat{\phi }\left(r\right)-1\right)}^{2}, \end{array}$$

where $f_{0}$ is the unknown free energy per unit volume in the reference state, in units of $k_{B}T$. We clarify that $f_{0}$ is the excess free energy, i.e., the translational-entropy contribution $f_{\mathrm{id}}\left(\rho_{0}\right)=\left(\rho_{0}/N\right)\ln\left(\nu_{0}\rho_{0}/N\right)$ is omitted ($\nu_{0}$ stands for a thermal “volume”). Considering in Equation (6), an excess free energy functional is consistent with the fact that segmental coordinates are explicitly present in the partition function (see later Equation (A1)). Accordingly, $\mu_{0}\left(\rho_{0}\right)$ is the excess chemical potential of a polymer chain in the reference state and $k_{T}$ the isothermal compressibility. For compactness, in Equation (6), the coefficient involving $k_{T}$ is replaced by $\kappa \rho_{0}$, where *κ* is now a compressibility parameter.

The anisotropic part is approximated in a Maier–Saupe form,

(7) $$\frac{H_{\mathrm{nem}}}{k_{B}T}=-\frac{\rho_{0}\upsilon }{3}\int dr\hat{S}\left(r\right):\hat{S}\left(r\right).$$

The positive parameter *υ* sets the strength of orientation-coupling between two segments under consideration and encourages liquid-crystal formation.

Equation (6) reduces to the Helfand and Tagami constraint [59], commonly employed in a field theory as a simple method to penalize the density fluctuations. The first two trivial terms, a background free energy and chemical potential contributions, are retained here to make the discussion of the thermodynamics more transparent from the perspective of comparison with experiments and computer simulations. They have no direct effects on the thermodynamics of the isotropic-nematic transition discussed below.

## 3. SCFT of Wormlike Polymers and the Thermodynamics of a Compressible MS Model

The basic idea is to approximate the interaction between particles by an external mean-field, now acting on a single wormlike chain. As illustrated in Figure 1d, the external field must contain both position- and orientation-dependencies, and the treatment of a wormlike system hence is mathematically more complicated in comparison with the treatment of a Gaussian-chain system [63]. Starting from the basic Hamiltonian for wormlike polymer configurations in (1) and interaction potential in (6) and (7), in Appendix A, we derive the Helmholtz free energy of a spatially non-uniform nematic state at a mean field level. Here, in this section, we summarize briefly the final free energy that is useful for calculating the thermodynamics of a bulk system where the density and nematic ordering are uniform across the entire system, as a special example.

In this case, we can define an overall volume fraction,

(8) $$\phi =nN/V\rho_{0},$$

and orientational order parameter:

(9) $$S=\frac{1}{2}\langle 3\cos^{2}\theta -1\rangle ,$$

where *θ* is the angle that a molecular segment makes with respect to the nematic director. As presented in Appendix B, we can deduce the free energy in reduced units, given by:

(10) $$\begin{array}{cc} \Delta \tilde{F}\left(\phi ,S;M\right) & =\tilde{F}-{\tilde{F}}^{\prime }\equiv \frac{N\beta \Delta F}{V\rho_{0}}\\ & =\phi \ln\left(\frac{\phi }{Q_{b}}\right)+\frac{N\kappa }{2}{\left(\phi -1\right)}^{2}\\ & -\frac{N\upsilon }{2}S^{2}\phi^{2}+\left[\ln\left(\frac{\nu_{0}\rho_{0}}{N}\right)+\beta \mu_{0}\right]\left(\phi -1\right)+\frac{3}{2}MS\phi \end{array}$$

where a reference free energy has been introduced,

(11) $$\begin{array}{c} {\tilde{F}}^{\prime }=\ln\left(\frac{\nu_{0}\rho_{0}}{N}\right)+\frac{Nf_{0}}{\rho_{0}}. \end{array}$$

The variable *M* is an external field conjugated to *S*, which under the saddle-point condition is given by M=(2/3)NυSϕ. The calculation of the single chain partition function $Q_{b}$ in the mean field *M* is the most complicated part of the model. We refer to the Appendices for the detailed procedure.

Based on the derivative of the free energy, the reduced pressure difference is:

(12) $$\begin{array}{cc} \Delta \tilde{P}\left(\phi \right) & \equiv \tilde{P}-{\tilde{P}}^{\prime }\\ & =\phi -1+\frac{N\kappa }{2}\left(\phi^{2}-1\right)-\frac{N\upsilon }{2}S^{2}\phi^{2}. \end{array}$$

where the reference pressure itself:

(13) $${\tilde{P}}^{\prime }=1-Nf_{0}/\rho_{0}+\beta \mu_{0}$$

is the pressure of the reference bulk isotropic polymer state. We can also find a reduced chemical potential:

(14) $$\begin{array}{c} \beta \Delta \mu \left(\phi \right)\equiv \beta \mu -\beta \mu^{\prime }=\ln\left(\frac{\phi }{Q_{b}}\right)+N\kappa \left(\phi -1\right). \end{array}$$

The reference chemical potential has the form:

(15) $$\beta \mu^{\prime }=\ln\left(\frac{\nu_{0}\rho_{0}}{N}\right)+1+\beta \mu_{0}.$$

In this case, both ${\tilde{P}}^{\prime }$ and $\beta \mu^{\prime }$ have been taken as the thermodynamic quantities of the reference state, which are functions of the density $\rho_{0}$.

The overall orientational order parameter, averaged over all segments in the system, is defined as:

(16) $$S=\langle P_{2}\left(\cos\theta \right)\rangle$$

where we assume that the uniaxial nematic director aligns with the *z*-axis of the laboratory frame and *θ* is measured from this axis. Both *S* and $Q_{b}$, which depends on *S*, must be determined self-consistently, through solving (A12), (A13), (A15) and (A16), according to the procedure described in Appendix B.

Near the transition point, there are two branches of the free energy, pressure and chemical potential, corresponding to an isotropic phase (I) and nematic phase (N). The phase transition point has the properties,

(17) $$\beta \Delta \mu_{I}\left(\phi_{I}\right)=\beta \Delta \mu_{N}\left(\phi_{N}\right).$$

and:

(18) $$\Delta {\tilde{P}}_{I}\left(\phi_{I}\right)=\Delta {\tilde{P}}_{N}\left(\phi_{N}\right).$$

Solving the last two equations determines the coexistence condition of the two states and yields the coexistence volume fractions $\phi_{I}$ and $\phi_{N}$.

## 4. Density Gap of a Physical System at the Transition

The theory involves a number of parameters; we stress that only a few combinations of them are physically relevant quantities. In fact, these combinations can be employed to establish a connection between the mesoscopic model with a real material. Such combinations were referred to in [64] as “invariants”.

We start with the flexibility of a wormlike polymer chain. There are two characteristic length scales of a polymer chain modelled here. Both *L* and $L_{p}$ are well-defined, measurable physical length scales. The unique combination is the flexibility $L/L_{p}$, which shows up in Equation (1) or, in the actual calculation of the conformational properties, Equation (A12). For example, poly(3-alkylthiophenes) (P3AT) at elevated temperatures is characterized [65] by $L_{p}≃1.5$–3nm. In experiments that target LC behaviour, the number of repeat units commonly ranges from a few tens to hundreds, which is equivalent to L≃12–50nm [9]. This motivates our choice throughout the paper, $L/2L_{p}≃2.7848$, representing a case of low-molecular-weight, semiflexible P3AT (on the order of 30 thiophenes). Of course, other values of $L/2L_{p}$ can be used in our formalism, but are not discussed here.

Another unique combination is the compressibility parameter Nκ, which appears in Equations (12)–(14), as well as in the reduced free energy. To explore effects of compressibility, several values ranging from Nκ=80–1280 are selected for the theoretical study in the next section. What values of isothermal compressibility do these reduced values correspond to? As an approximation, we take T=373K and ϕ≈1 and assume the system is in an isotropic state where S=0. The chain density, n/V, can be estimated assuming that the real polymers (i.e., the short P3AT) have a density on the order of 1g/cm${}^{3}$ and molecular weight $5\times 10^{3}\;$g/mol. Through Equation (A20), we can hence estimate that the chosen range of Nκ leads to compressibilities between $k_{T}≃2\times 10^{-8}\;$Pa${}^{-1}$ and $1.26\times 10^{-9}\;$Pa${}^{-1}$. The smallest $k_{T}$ is representative of a situation encountered in the compressible mesoscopic model [58,60,66], while the largest is comparable to the compressibilities of real materials.

Within the model, the other unique parameter is the combination υN, which serves as a free parameter in the examples presented below, a large value of which drives the system to a nematic state. Both in defining the mean-field, Equation (A15) and typical thermodynamic expressions in (12)–(14), the Maier–Saupe coefficient always appears as a combination Nυ. One can argue that both Nκ and Nυ can have density and temperature dependencies. Here, we assume that in a thermotropic system Nυ is a temperature-dependent parameter that drives the phase transition, while Nκ has a much weaker temperature dependence in the transition regime. Generally, one can assume [9] that *υ* has a Flory–Huggins-like temperature dependence, υ=A+B/T, where *A* and *B* are constants, but this assumption is not required here.

**Figure 2:**
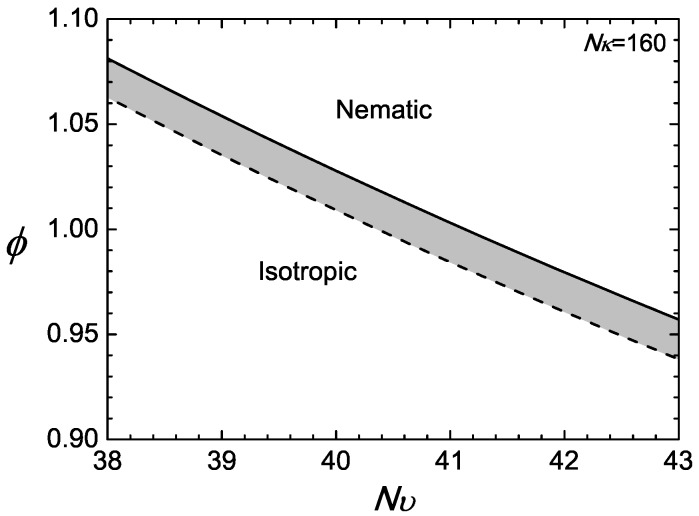
Example of a phase diagram for a compressible system with Nκ=160. The phase space is divided into two regimes: nematic above the nematic volume fraction $\phi_{N}$ represented by the solid line; and isotropic below the isotropic volume fraction $\phi_{I}$ represented by the dotted line.

In the example presented here, we first specify $L/2L_{p}=2.7848$ and then demonstrate the isotropic-nematic transition properties by letting Nκ=160. For every specified Nυ, the first-order transition point is determined by solving (18) and (17) simultaneously for $\phi^{N}$ and $\phi^{I}$. The numerical results are displayed in Figure 2, where a jump in the volume fractions between the nematic $\phi^{N}$ (solid) and isotropic $\phi^{I}$ (dotted) is visible. The figure can be viewed as phase diagrams; above $\phi^{N}$, the nematic state is stable, and below $\phi^{I}$, the isotropic state is stable; the shaded region is a co-existence region.

## 5. The Isobaric Properties

To demonstrate the crossover from a compressible system to an incompressible system, we examine the coexistence region under different magnitudes of the compression parameter Nκ. We first take Nκ=160 as an example.

**Figure 3:**
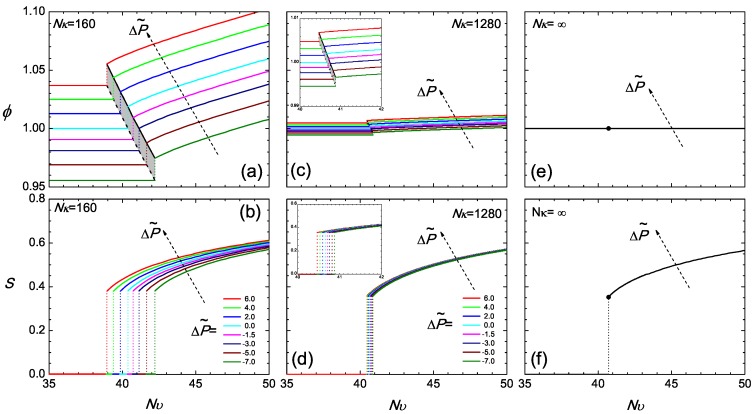
The isobaric volume fraction *ϕ* (**a**,**c**,**e**) and the isobaric orientational order parameter *S* (**b**,**d**,**f**), as a function of Nυ across the transition region for Nκ=160,1280 and ∞. The value of the reduced $\Delta \tilde{P}$ is specified in Plot (**b**). In this phase diagram, the coexistence region is shaded.

Regarding Nυ as the main temperature-dependent parameter, we plot various isobaric curves in Figure 3a according to the current model, by fixing $\Delta \tilde{P}\left(\phi ,N\upsilon \right)$ at various values and directly solving (12) for *ϕ* as a function of Nυ. To obtain the isotropic branch where S=0, one simply inverts (12), and *ϕ* is a constant over the the entire branch. The nematic branch requires the solution of the SCFT model, where a *ϕ*-dependent *S* needs to be considered in this equation. Across Nυ corresponding to the transition, the density jump, which is also illustrated in Figure 2, can be viewed in this diagram. The narrow shaded region represents the coexistence region of the two states.

It is instructive to estimate the change in the pressure, $\Delta \tilde{P}$, required to slightly change the density in the isotropic state. From Equation (13) assuming that *ϕ* changes in the vicinity of unity, we can deduce $\Delta \tilde{P}\approx N\kappa \left(\phi -1\right)$. Even for Nκ=160 (which is an order of magnitude smaller than in the case of a real material), we see that the 1% change in *ϕ* from ϕ=1 in the isotropic state corresponds to an approximate value $\Delta \tilde{P}∼1.6$. This amounts to a $P-P_{0}\approx 10\times 10^{5}$ Pa, after the scaling factor $\beta PN/\rho_{0}$ is estimated using the parameters provided in Section 4. Approximately, this is about a ten-time increase with respect to atmospheric pressure (at room-temperature). Hence, the current regime of compressibility describes systems where the density increases only by a small percent, although the pressure is substantially raised from the reference state.

The isotropic-nematic density gap at the transition, in terms of the volume fraction, becomes smaller in a system with relatively large Nκ. Two features exist in Figure 3c at Nκ=1280. In a similar range of reduced pressure, as considered for Nκ=160, the density gap tremendously reduces, and the volume fraction varies in the vicinity of unity; the range of the transition temperatures for different pressures reduces down to a small region. It should be noted that the free energy term associated with Nκ can be mathematically viewed as a term penalizing deviations of *ϕ* from unity. In a system with a large Nκ, the volume fraction is forced to become unity, regardless of whether it is in the isotropic or nematic state. In this scenario, the reduced pressure, (12), and chemical potential, (14), are essentially dominated by the Nκ term, so that the equilibrium conditions (17) and (18) become automatically satisfied in the leading order. Next to the leading terms, the small variations produce small adjustments in the volume fractions of the two states from unity.

The density jump at the isotropic-nematic transition is fairly sensitive to the compressibility, as demonstrated above. In contrast, the value of the order parameter at the transition point depends only weakly on Nκ and $\Delta \tilde{P}$, shown in Figure 3b,d,f, for the fairly broad range of values investigated here. This result can be rationalized by the consideration that the bulk propagator equation, (A12) (coupled with the ordering field in (A15)), the orientational order parameter defined in (A16) and the bulk single-chain partition function $Q_{b}$ defined in (A13) depend explicitly only on the strength of the mean field. An increase in the density is compensated by a decrease in the strength of interactions, Nυ, required for bringing up the transition (similar argument holds when density is decreased). Therefore, the strength of the mean field at the transition point is similar for the different systems, being proportional to Nυϕ (see (A15)). As a consequence, one obtains a very similar jump of the orientational order parameter.

This physical picture is more transparent in the asymptotic limit of Nκ=∞, which describes an incompressible system with a constant ϕ=1 across the isotropic and nematic regions. All isobaric curves now collapse into two single curves: a constant ϕ=1 in Figure 3e and a universal *S* curve in Figure 3f in the nematic state. All signatures of pressure dependence are now erased. Theoretically, this is an interesting limit, as the two equilibrium equations, (17) and (18), are automatically satisfied and no longer required to be considered; one is left with the two free energy branches, which cross each other at the transition point; the crossing becomes the only condition required to determine the isotropic-nematic transition point in Nυ.

It is this asymptotic case where the previous studies have been carried out. To solve the SCFT model for the isotropic-nematic transition, Spakowitz and Wang proposed an exact theoretical approach based on the Laplace-transformed partition function of a wormlike chain [67]. By examining the crossing of the isotropic and nematic branches of the free energy, Jiang et al numerically located the transition point in Nυ and the jump of the orientational order parameter *S* at the transition [45]. Letting $L/2L_{p}=2.7848$ yields Nυ=40.704 and S=0.353, exactly matching the transition point presented in Figure 3f. In an earlier attempt, Rusakov and Shliomis used a Landau-de Gennes free energy expansion from the same theory [42]; substituting $L/2L_{p}=2.7848$ into their results gives S=0.2165 at a transition point Nυ=43.946. While the former is apparently an underestimate, the latter agrees well with the transition point determined from the more precise numerical work. These studies used a free energy model for incompressible wormlike polymers; formally, such a model can be obtained from the one described in the current work, in the limit κN→∞.

Returning to the general Nκ case, we note that the value of *S* at the transition point is close to 0.35 approximately, which is within the range of 0.30–0.7 determined in conventional low molecular weight liquid crystal systems [68,69,70,71,72]. Recently, the liquid crystalline ordering for poly(3-hexylthiophene) (P3HT) (a basic material for research in organic electronics) was investigated experimentally [73]. The nematic order *S* was observed to increase to 0.35, depending on the preparation conditions of the polymer solution. It is encouraging to see that the theoretically calculated *S* and the experimentally determined *S* are in the same range.

## 6. The Free Energies

**Figure 4:**
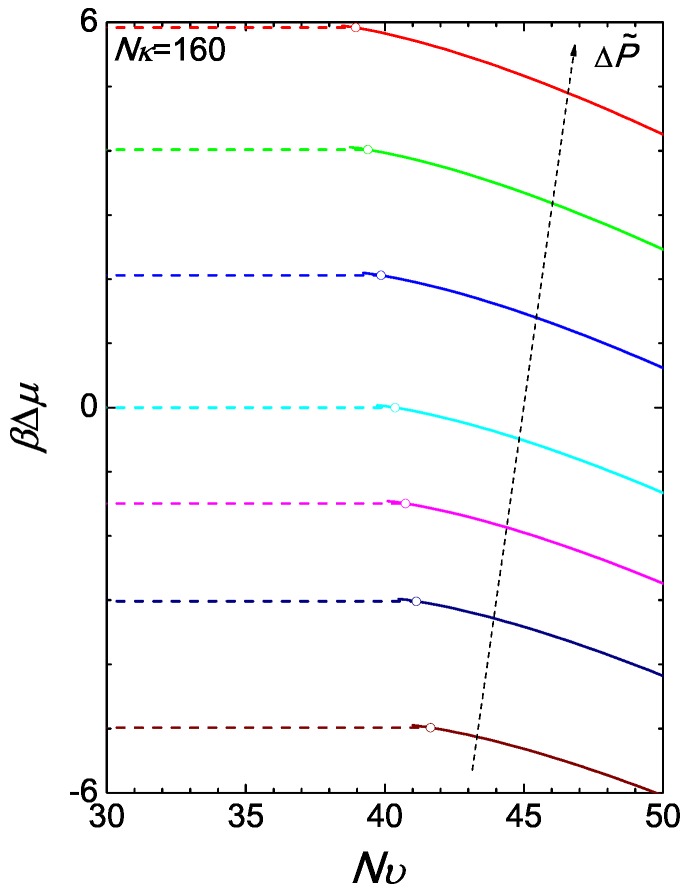
The two branches of the reduced chemical potential difference βΔμ. The isotropic-nematic transition can be inferred from the crossing of the isotropic and nematic branches of the Gibbs free energies per chain (equivalent to the chemical potentials plotted here). The colour of a curve corresponds to the pressure difference defined in Figure 3.

The isotropic-nematic transition considered here is a first-order transition. As such, the Gibbs free energies of the two branches of the isobaric curves as a function of pressure and temperature are expected to cross each other at the phase transition. Following the definition: the Gibbs potential reads:

(19) βG/n=βF/n+βPV/n=βμ

where in terms of *ϕ* and Nυ the right-hand side is given by (A17). Using the chemical potential of the reference state, (15), we have illustrated the chemical potential difference in Figure 4 for the case of Nκ=160. The isotropic branch is produced simply by ln(ϕ)+Nκ(ϕ−1) where *ϕ* is a constant over Nυ as shown in Figure 3a. At a given $\Delta \tilde{P}$, at the transition point, the Gibbs free energies of the two branches cross each other at a single point.

We return to the canonical ensemble here with ϕ,Nυ as the basic parameters. In a system where the total n/V is fixed, relative to the reference density, the overall *ϕ* is also fixed. This can happen in, for example, a computer simulation, where *n* polymer chains are placed in a volume *V*. The phase diagram in Figure 2a can then be viewed from a different perspective: for a fixed *ϕ*, there exists a lower bound $N\upsilon_{I}$ and a upper bound $N\upsilon_{N}$, between which the system is found in a coexistence regime. The phase transition is induced by varying Nυ, which is a much easier adjustable parameter.

**Figure 5:**
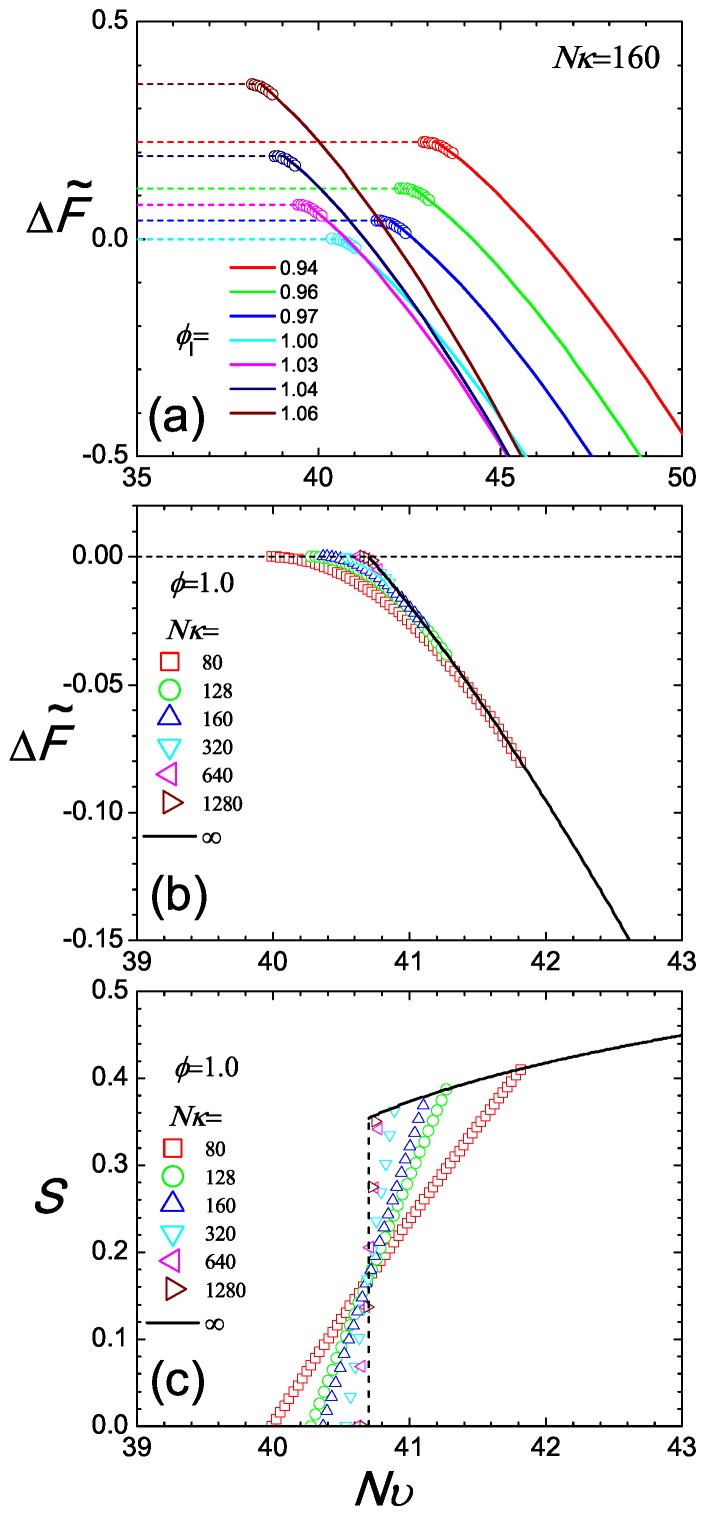
Two perspectives of the isotropic and nematic branches of the Helmholtz free energy difference plotted in (**a**,**b**) and the orientational order parameter in the coexistence regime (**c**). In Plot (**a**), we examine a system with Nκ=160; each set of data is plotted according to a specified volume fraction, ϕ=0.94,0.96,0.97,1.00,1.03,1.04,1.06, where the isotropic branch is shown with a dashed line and the nematic branch with a solid curve. Circles represent the co-existence region where the free energy difference follows the tangent rule and connect the two branches. In Plots (**b**,**c**), we fix the total, averaged *ϕ* and observe the two free energy branches (again dashed and solid curves for isotropic and nematic states, respectively) and *S* as functions of the temperature-like parameter Nυ. The free energy difference and *S* in the coexistence region are calculated from the mixing rule and plotted by symbols as a function of Nυ, for various degrees of compressibility Nκ=80 (square), 128 (circle), 160 (up-triangle), 320 (down-triangle), 640 (left-triangle), 1280 (right-triangle), ∞ (solid line).

We examine the Helmholtz free energy by raising Nυ from the isotropic state. According to (10), the isotropic branch of the Helmholtz energy difference can be written as:

(20) $$\begin{array}{c} \Delta \tilde{F}\left(\phi ,0\right)=\phi \ln\left(\phi \right)+\frac{N\kappa }{2}{\left(\phi -1\right)}^{2}+\left[\ln\left(\frac{\nu_{0}\rho_{0}}{N}\right)+\beta \mu_{0}\right]\left(\phi -1\right), \end{array}$$

which is a constant over Nυ for a given *ϕ*. Note that due to the normalization constant introduced in (A13), $Q_{b}=1$ in an isotropic state. Here, the difference is made with respect to the free energy of the reference system defined in Equation (11). Removing the last term, which for every Nυ is constant for a system with fixed *ϕ*, we have plotted the lower branch of the free energy in Figure 5a for various values of $\phi_{I}$ as horizontal dashed lines. This is valid below $N\upsilon_{I}$ for a given *ϕ*.

Beyond $N\upsilon_{N}$, the free energy follows the nematic branch. In this case, $\Delta \tilde{F}$ is obtained from (10) by solving numerically the SCFT equations (see Appendix). The results are plotted in Figure 5a, where again, the last constant term is omitted. As Nυ increases, the free energy significantly decreases, dominated by the negative −Nυ term in this expression.

The regime $N\upsilon_{I}<N\upsilon <N\upsilon_{N}$ is where both isotropic and nematic regions coexist in the system for a fixed *ϕ*. Ideally at thermodynamic equilibrium, these two regions are separated by a single interface; however, in metastable systems, it is possible that multiple isotropic and nematic domains are formed. We now assume the coexistence of the isotropic (I) and nematic (N) domains, at a particular Nυ. Each type of domain contains $n_{i}$ polymers occupying a subvolume $V_{i}$ where i=I,N and is characterized by its own volume fraction,

(21) $$\phi_{I}\equiv n_{I}N/V_{I}\rho_{0}\;\;\;\;\;\phi_{N}\equiv n_{N}N/V_{N}\rho_{0}.$$

Two constraints must be considered here:

(22) $$V_{I}+V_{N}=V$$

and:

(23) $$n_{I}+n_{N}=n.$$

The parameters $n_{I}$, $n_{N}$, $V_{I}$ and $nV_{N}$ are adjusted to minimize the total free energy.

Hence, we have two constraints:

(24) $$f_{I}+f_{N}=1$$

and:

(25) $$\phi_{I}f_{I}+\phi_{N}f_{N}=\phi$$

where $f_{I}$ and $f_{N}$ (both positive) are the volume fractions of the two phases, and *ϕ* is the total volume fraction in the system, specified according to our assumption in this section. For fixed $\phi_{I}$ and $\phi_{N}$, these two equations have valid solution for $f_{I}$ and $f_{N}$ only in the regime $\phi_{I}<\phi <\phi_{N}$,

(26) $$f_{I}=\frac{\phi_{N}-\phi }{\phi_{N}-\phi_{I}}$$

and

(27) $$f_{N}=\frac{\phi -\phi_{I}}{\phi_{N}-\phi_{I}}.$$

The free energy difference in the coexisting regime can also be determined as:

(28) $$\Delta \tilde{F}=f_{I}\Delta {\tilde{F}}_{I}\left(\phi_{I}\right)+f_{N}\Delta {\tilde{F}}_{N}\left(\phi_{N}\right)$$

where ${\tilde{F}}_{I}$ and ${\tilde{F}}_{N}$ are the isotropic and nematic free energies at the phase boundaries for the Nυ value under examination. This procedure, known as the tangent construction, is usually used for calculating the free energy of the co-existence region.

The compressibility Nκ plays a crucial role in controlling the size of the coexistence region. We have already seen this in Figure 3, where we compare the density gaps between two cases, Nκ=160 and Nκ=1280. Here, we can further illustrate this point by taking a typical case, ϕ=1, and examining the free energy plot, Figure 5b for various Nκ. All branches share the same free energy profile in the pure nematic regime because the total segment density of the system is fixed. For small compressibility, e.g., Nκ=80,128,160, the free energy in the coexistence regime exhibits a relatively gentle transition connecting the isotropic and nematic states. Using the same mixing rule, we demonstrate the corresponding *S* in the coexistence region, where *S* is seen to continuously vary from $S_{N}$ to zero for Nκ≠∞ cases. Connecting with $S_{N}$, there is a sharp turn at $N\upsilon_{N}$, expected in the thermodynamic limit. However, in a finite system, this sharp turn can become smooth.

A similar interaction Hamiltonian can be implemented in the NVT ensemble in a computer simulation. The isotropic-nematic transition is then conveniently observed by varying the temperature-like variable Nυ. A simulation-deduced Helmholtz free energy (if possible) and *S* would have similar features as in Figure 5b, where the connection between the isotropic and nematic branches in the coexistence region appears smooth in free energy (apart from Nκ→∞) and *S* continuously vary from $S_{I}=0$. The smoothness should not be confused with a typical second-order transition, for which the free energy is continuously connected through Nυ, up to and including the first derivative; a second-order transition could be misidentified by these plots.

## 7. Remarks: A Particle-Based Perspective

So far, we focused on the field-theoretical implementation of the compressible MS model defined in Section 2. To demonstrate that the conclusions drawn from SCFT calculations are indeed relevant for mesoscopic computer simulations of polymer liquid crystals, we discuss in this section the particle-based interpretation of the model.

In a particle-based representation, the total energy from pairwise non-bonded interactions between coarse-grained segments can be written as:

(29) $$\begin{array}{c} \frac{H_{\mathrm{nb}}^{\prime }}{k_{B}T}=\frac{N^{2}}{2}\sum_{i=1}^{n}\sum_{j=1}^{n}\int_{0}^{1}\int_{0}^{1}dtdt^{\prime }U_{}\left[r_{ij}\left(t,t^{\prime }\right);u_{i}\left(t\right),u_{j}\left(t^{\prime }\right)\right] \end{array}$$

The potential *U* (in units of $k_{B}T$) represents the interaction between two polymer segments of length Ndt and $Ndt^{\prime }$, respectively. The distance between these two segments is given by $r_{ij}\left(t,t^{\prime }\right)$, while $u_{i}\left(t\right)$ and $u_{j}\left(t^{\prime }\right)$ are their unit tangent vectors (Figure 1c). We note that expressing $H_{\mathrm{nb}}^{\prime }/k_{B}T$ as above includes immaterial contributions from self-interactions. Taking an expansion of the potential function in terms of the Wigner rotation matrices [74,75], we obtain:

(30) $$U\left(r_{12};u_{1},u_{2}\right)=U_{0}\left(\right|r_{12}\left|\right)+U_{1}\left(\right|r_{12}\left|\right)\left[Q\left(u_{1}\right):Q\left(u_{2}\right)\right]+\ldots$$

which can be inserted into (29). Here, we introduced the shorthand notation 1 for the *t*-th segment of the *i*-th chain and 2 for the $t^{\prime }$-th segment of the *j*-th chain.

To compare with the functional-based representation of non-bonded interactions introduced in Section 2, we write:

(31) $$U_{0}\left(\right|r_{12}\left|\right)=\frac{\kappa }{\rho_{0}}{\bar{U}}_{0}\left(\right|r_{12}\left|\right),$$

and:

(32) $$U_{1}\left(\right|r_{12}\left|\right)=-\frac{1}{3}\frac{\upsilon }{\rho_{0}}{\bar{U}}_{1}\left(\right|r_{12}\left|\right).$$

The functions ${\bar{U}}_{0}$ and ${\bar{U}}_{1}$ are normalized to unity. Their coefficients, *κ* and *υ*, control the strength of the two interactions. One can regard both ${\bar{U}}_{0}$ and ${\bar{U}}_{1}$ as three-dimensional delta-functions. Substituting then Equation (30) into Equation (29) recovers terms that are quadratic in $\hat{\phi }\left(r\right)$ and $\hat{S}\left(r\right)$ (cf. Section 2). Formally, terms that are linear in $\hat{\phi }\left(r\right)$ or constant (cf. Equation (6)) are not recovered. Although these terms affect the equation of state in the reference state (see Equations (13) and (15)), they have no bearing on the thermodynamics of the isotropic-nematic transition, determined by Equations (17) and (18).

To perform computer simulations [56,57,60], one must (i) substitute the continuum wormlike chain model with its discrete counterpart and (ii) “regularize” the delta-functions by replacing them [27] with “soft-cores” (normalized to unity) of finite interaction-range, *σ*. These functions are typically selected to effectively represent the results from a simple heuristic treatment of the integrated-out microscopic degrees of freedom. For conjugated polymers, *σ* is on the order of a side-chain span [56,58,60]. After these transformations, the compressible MS model can serve as a framework for standard computer simulations. Importantly, to facilitate direct comparison with simulations [56], the SCFT formalism presented in this study can be employed in the framework of the discrete wormlike chain model. In this case, partial enumeration schemes can be used for the numerical solution of SCFT [56,76,77,78].

## 8. Summary

This paper concerns systems of nematic polymers, described through a compressible Maier–Saupe model combined with a wormlike-chain representation of polymer architecture. The model is suitable for field-theoretical developments and computer simulations. Here, the statistical mechanics of the model is considered within the SCFT approximation focusing on the isotropic-nematic phase transition. Among the thermodynamic properties presented, we highlight the change of the transition properties and the range of the isotropic-nematic coexistence region when the compressibility parameter varies.

Though this paper is theoretical, major attention is paid to connect with real experimental systems. To this end, we showcase an example where the polymer length/persistence ratio is set at an experimentally relevant value $L/2L_{p}=2.7848$; such a polymer is semiflexible. We consider a range of compressibilities representative of actual polymeric materials. The formalism itself is valid for a diverse range of the flexibility parameter $L/L_{p}$: from rod-like where $L/L_{p}\ll 1$ to coil-like where $L/L_{p}\gg 1$. Therefore, our approach can be used to study other real materials that can be represented through a compressible MS model.

It is interesting to note that within the liquid-crystal community, a temperature-dependent model is often referred to as a thermotropic system, whereas a temperature-independent model lyotropic. A typical lyotropic model stems from the work of Onsager [79], who originally studied rigid rods as a coarse-grained model for molecules, interacting with steric repulsive potentials only. Assuming that the segments are cylinder-like and have a non-vanishing excluding diameter, Onsager calculated the orientational-dependent second-virial kernel through a Mayer expansion. There is a single coefficient that represents the magnitude of the excluded-volume interaction, directly proportional to the diameter of the modelled cylinders. The generalization of the Onsager kernel to handle the segment-segment interactions in PLC theory is due to Khokhlov and Semenov [38]. Indeed, a SCFT theory can be developed to handle the thermodynamics of a compressible PLC system originating from the excluded-volume only [38,39,40]. An expansion of the Onsager kernel in terms of Wigner matrices leads to two leading terms of the form presented in Equation (29). Of course, the two coefficients, *κ* and *υ* (both can be shown to be positive), are now related and both have the meaning of an excluded-volume. As they are temperature independent, the potential in Equation (29) can be also used to describe a lyotropic system. Thus, this expression can serve as a general basis for modelling PLC systems, regardless of the origin of the physical mechanism that leads to the liquid-crystal ordering.

Generally, the softness of potentials in a particle-based simulation does not automatically imply large compressibility, since it can be compensated [25,80] by subtle features in pairwise interactions (such as long-range tails). However, heuristically introduced potentials lack these features, and mesoscale models are too compressible. Our SCFT calculations demonstrate that for compressibilities typical for mesoscale simulations, certain properties of the isotropic-nematic transition are enhanced comparing to real materials. Some examples are the substantial change of volumetric properties upon transition, broader coexistence region, and increased sensitivity of the location of the transition to pressure. Qualitatively, the predictions of SCFT and simulations can differ even when considering exactly the same MS model, due to approximations in the former. For instance, recently it was demonstrated [56] that the comparison of free energies in SCFT with their counterparts in mesoscopic simulations based on the MS model is affected by local correlations, neglected in the former. We do not expect, however, that the approximations intrinsic to the current SCFT treatment affect qualitatively the predicted trends.

The main ideas developed in the current paper can be easily generalized to multi-component polymers, adding a tool that is useful for studying compressible systems. A parallel theoretical development for incompressible multi-component semiflexible-polymers (such as block copolymers), which includes the consideration of directional ordering, was recently discussed in a perspective paper [81]. The features for describing spatial inhomogeneities are already built-in according to the calculation scheme presented in our Appendix A. These features are important for the determination of the crystallographic structures resulting from micro-phase separation of copolymers with wormlike blocks.

## Appendix A. SCFT of the Impressible MS Model for Liquid Crystal Polymers

In this Appendix, we lay out the main structure of SCFT on the basis of the Hamiltonian models presented in Section 2. We consider a general case for a spatially inhomogeneous and directionally ordered system, although the application in the text is mainly for a system with no positional dependence.

To calculate the free energy, we consider the partition function of the system,

(A1) $$Z=\frac{1}{n!}\int \prod_{i=1}^{n}DR_{i}\exp\left(-\frac{H_{b}}{k_{B}T}-\frac{H_{\mathrm{nb}}}{k_{B}T}\right),$$

where $\int DR_{i}$ samples all possible configurations of the *i*-th chain according to the statistical weight given by the Boltzmann factor. The spatial and orientational variations of the system are characterized by the configuration-dependent (2) and (4). Note that the density fraction $\hat{\phi }$ defined is normalized by (3). In a compressible system, the local $\hat{\phi }\left(r\right)$ can be beyond or below one, as the local density varies around the reference value $\rho_{0}$. The interaction Hamiltonian in Section 2 can then be rewritten by:

(A2) $$\frac{H_{\mathrm{nb}}}{k_{B}T}=\rho_{0}\int dr\left[\frac{\kappa }{2}{\hat{\phi }}^{2}\left(r\right)-\frac{\upsilon }{3}\hat{S}\left(r\right):\hat{S}\left(r\right)+\alpha \hat{\phi }\left(r\right)+\gamma \right],$$

where two constants, *κ* and *υ*, separately control the magnitudes of the two interaction terms. We use constants *α* and *γ* in the Appendices, and these constants are related to those in Section 2 by $N\alpha =\beta \mu_{0}-N\kappa$ and $N\gamma =Nf_{0}/\rho_{0}-\beta \mu_{0}+N\kappa /2$.

Taking this particle-based representation of the partition function, in order to make the Hubbard–Stratonovich transformation, we introduce two conjugate fields to $\hat{\phi }$ and $\hat{S}$, a scalar W(r) and a tensor M(r), respectively. The saddle-point approximation is proposed here to pick up the most optimal free energy contribution among all configurations. For the mathematical construction of SCFT, please refer to [35] on a theory based on the Gaussian model, or [63] on a theory based on the WLC model. We finally arrive at the free energy of the system,

(A3) $$\begin{array}{cc} \beta F & \left(\phi ,S;W,M\right)=-\ln Z\approx n\ln\left(\frac{n\nu_{0}}{QV}\right)+\\ & +\rho_{0}\int dr\left[\frac{\kappa }{2}\phi^{2}\left(r\right)-\frac{\upsilon }{3}S(r):S(r)+\alpha \phi (r)+\gamma \right]\\ & -\rho_{0}N^{-1}\int drW\left(r\right)\phi \left(r\right)+\rho_{0}N^{-1}\int drM\left(r\right):S\left(r\right), \end{array}$$

where $\nu_{0}$ is the thermal volume introduced here for dimensional purpose, which shifts the chemical potential zero only. The approximation sign here represents the fact that an approximation has been made at the mean-field level. The free energy expression is a functional of four functions ϕ(r),S(r),W(r) and M(r). The canonical-ensemble-averaged, r-dependent quantities,

(A4) $$\phi \left(r\right)\equiv \langle \hat{\phi }\left(r\right)\rangle$$

and

(A5) $$S\left(r\right)\equiv \langle \hat{S}\left(r\right)\rangle ,$$

are used above. The volume fraction ϕ(r) is the ratio between the local density and $\rho_{0}$. The expression contains a single-chain partition function *Q* in the combined external fields W(r) and M(r). It can be computed from the solution of the reduced Green’s function q(r,u;t), similar to the Feynman–Kac formula in the path-integral description of quantum mechanics, [82]

(A6) $$Q=\frac{1}{4\pi V}\int dr\int du\;q\left(r,u;t=1\right).$$

The reduced Green’s function, q(r,u;t=1), often called a propagator in polymer theories, satisfies the following modified diffusion equation:

(A7) $$\begin{array}{c} \frac{\partial }{\partial t}q\left(r,u;t\right)=\left[\frac{L}{2L_{p}}\nabla_{u}^{2}-Lu\cdot \nabla_{r}-W\left(r\right)+M\left(r\right):Q\left(u\right)\right]q\left(r,u,t\right) \end{array}$$

subject to the initial condition q(r,u,0)=1. The reduced arc-variable *t* has a range of [0,1], covering one end of the polymer to another.

The partition function *Q* is a functional of both W(r) and M(r) at this stage. The saddle-point condition leads to:

(A8) $$\phi \left(r\right)=\frac{\rho }{4\pi \rho_{0}Q}\int du\int_{0}^{1}dtq\left(r,u;t\right)q\left(r,-u;1-t\right)$$

and

(A9) $$S\left(r\right)=\frac{\rho }{4\pi \rho_{0}Q}\int du\int_{0}^{1}dtQ\left(u\right)q\left(r,u;t\right)q\left(r,-u;1-t\right),$$

where *ρ* is the overall monomer density in the system.

## Appendix B. Free Energy of a Spatially Uniform State

In a bulk (b) state, there is no position-dependence. We then drop all r-dependence in the formalism presented in the last Appendix. When the entire system is in a completely uniform state,

(A10) $$\phi \left(r\right)=\phi \equiv \rho /\rho_{0},$$

as required by the normalization condition in (3). In addition, we choose the *z*-axis as the principal director of the nematic state. The tensor S is therefore a diagonalized and traceless matrix satisfying $S_{xx}=S_{yy}=-S_{zz}/2$; in this case, only one of the three components, $S_{zz}$, which we denote as a scalar Sϕ, is independent. The matrix M has the same symmetry properties and has a zz-element *M*. The contraction between the tensors thus gives, $S:S=\left(3/2\right)S^{2}\phi^{2}$ and M:S=(3/2)MSϕ. All four functions in (A3) are now four variables.

The free energy expression can be largely simplified in a spatially uniform state:

(A11) $$\begin{array}{cc} \beta F(\phi ,S;W,M) & =n\ln\left(\frac{nv_{0}}{QV}\right)+nN\left(\frac{\kappa }{2}-\frac{\upsilon }{2}S^{2}\right)\phi \\ & -Wn+3nMS/2+\alpha Nn+\rho_{0}V\gamma . \end{array}$$

In an r-independent system, the partial differential equation in Equation (A7) can be transformed into:

(A12) $$\begin{array}{c} \frac{\partial }{\partial t}q_{b}\left(u;t\right)=\left[\frac{L}{2L_{p}}\nabla_{u}^{2}+\frac{3}{2}MP_{2}\left(\cos\theta \right)\right]q_{b}\left(u,t\right). \end{array}$$

where cosθ is the projection of the unit vector u on the *z*-axis, and $P_{2}$ is the second-rank Legendre polynomial. The partition function can now be factorized into:

(A13) $$Q=e^{-W}Q_{b}\equiv \frac{e^{-W}}{4\pi }\int du\;q_{b}\left(u;t=1\right),$$

where we have defined a *W*-independent $Q_{b}$. In reduced units, the free energy then becomes:

(A14) $$\begin{array}{cc} \tilde{F}\left(\phi ,S;M\right)\equiv \frac{N\beta F}{V\rho_{0}} & =\phi \ln\left(\frac{\phi \nu_{0}\rho_{0}}{Q_{b}N}\right)+\frac{N}{2}\left(\kappa -\upsilon S^{2}\right)\phi^{2}\\ & +N\alpha \phi +N\gamma +\frac{3}{2}MS\phi . \end{array}$$

Finally, the saddle-point condition gives us a simple form,

(A15) $$M=\frac{2}{3}N\upsilon S\phi ,$$

which needs to be inserted into the reduced partial differential Equation (A12) and free energy, Equation (A11). The order parameter is calculated from:

(A16) $$S=\frac{1}{4\pi Q_{b}}\int du\int_{0}^{1}dtP_{2}\left(\cos\theta \right)q_{b}\left(u;t\right)q_{b}\left(-u;1-t\right).$$

Equations (A12), (A13), (A15) and (A16) need to be solved self-consistently for a spatially homogeneous system. The result is a free energy function that depends on the density fraction *ϕ*.

We can readily calculate the chemical potential (after substituting the saddle-point condition into the free energy) by:

(A17) $$\begin{array}{c} \beta \mu \left(\phi \right)={\left[\frac{\partial \left(\beta F\right)}{\partial n}\right]}_{V,T}=\ln\left(\frac{\phi \nu_{0}\rho_{0}}{Q_{b}N}\right)+1+N\kappa \phi +N\alpha , \end{array}$$

where the *S*-dependence is implicitly contained in the partition function $Q_{b}$, and the pressure by:

(A18) $$\begin{array}{cc} \beta P & =-{\left[\frac{\partial (\beta F)}{\partial V}\right]}_{n,T}\\ & =\frac{n}{V}+\left(\frac{\rho_{0}\kappa }{2}-\frac{\rho_{0}\upsilon }{2}S^{2}\right)\phi^{2}-\rho_{0}\gamma . \end{array}$$

In terms of reduced units, the pressure is:

(A19) $$\tilde{P}\left(\phi \right)\equiv \beta PN/\rho_{0}=\phi +\left(\frac{N\kappa }{2}-\frac{N\upsilon }{2}S^{2}\right)\phi^{2}-N\gamma .$$

The reduced compressibility is then:

(A20) $$\begin{array}{cc} \frac{N}{\rho_{0}}\beta k_{T}^{-1} & =-\frac{NV}{\rho_{0}}{\left[\frac{\partial P}{\partial V}\right]}_{n,T}\\ & =\phi +\left(N\kappa -N\upsilon S^{2}\right)\phi^{2}. \end{array}$$
